# Pulmonary features and stage of disease in adult patients with hyper-IgE syndrome: a single-centre clinical study and literature review

**DOI:** 10.1186/s13023-025-03749-6

**Published:** 2025-06-03

**Authors:** Tiange Xie, Na Xu, He Zhao, Yingdong Han, Juan Wu, Hong Di, Min Peng, Ting Zhang, Hongwei Fan, Yun Zhang, Xuejun Zeng

**Affiliations:** 1 Department of Family Medicine & Division of General Internal Medicine, Department of Internal Medicine. Peking Union Medical College Hospital, Chinese Academy of Medical Sciences, State Key Laboratory of Complex Severe and Rare Diseases Peking, Union Medical College Hospital, No. 1 Shuaifuyuan, Dongcheng District, Beijing, 100730 China; 2 Department of Pulmonary and Critical Care Medicine, Chinese Academy of Medical Sciences, Peking Union Medical College Hospital, Peking Union Medical College, Beijing, China; 3 Department of Infectious Diseases, Chinese Academy of Medical Sciences, Peking Union Medical College Hospital, Peking Union Medical College, Beijing, China

**Keywords:** Hyper-IgE syndromes, *STAT3* gene, Primary immunodeficiency, Lung disease

## Abstract

**Background:**

The hyper-IgE syndromes (HIES) are a heterogeneous group of inborn errors of immunity-sharing manifestations including increased infection susceptibility, eczema, and raised serum IgE. Pulmonary complications are responsible for high morbidity and mortality rates in patients with HIES. This study examines the progression of pulmonary disease in adult patients with HIES and compares the subsequent findings with existing literature.

**Methods:**

Ten adult patients with HIES diagnosed at Peking Union Medical College Hospital (PUMCH) from January 2016 to October 2023 were included in this study. Diagnosis was confirmed using the National Institutes of Health (NIH) criteria and whole-exome sequencing. Clinical data on pulmonary disease progression, microbiology, imaging and histology were collected. A systematic literature review was conducted for comparison.

**Results:**

Recurrent pulmonary infections led to significant structural lung damage, with 90.0% (9/10) of patients developing bronchiectasis and pneumatocele. Early infections (0-10 years) were predominantly due to *Staphylococcus aureus* (80.0%,8/10), while later stages (6-22 years) showed a shift to more complex infections with *Aspergillus*/fungus (70.0%,7/10), *Mycobacterium tuberculosis* (50.0%, 5/10), and *Pseudomonas aeruginosa* (40.0%, 4/10). Imaging revealed extensive bronchiectasis and pneumatocele formation. Histological examinations demonstrated acute inflammation (40%, 2/5), granuloma formation (80%, 4/5), and eosinophilic infiltration (100%, 5/5). Comparatively, our findings are consistent with previous reports that suggest a higher incidence of pulmonary structural damage in patients with the signal transducer and activator of the transcription 3 (*STAT3*) mutations than in those with other gene variants. However, our cohort showed a faster progression from initial infection to structural damage, highlighting the need for early intervention.

**Conclusion:**

The progression of pulmonary disease in HIES patients underscores a critical three-step process: initial recurrent infections, development of structural lung damage, and subsequent reinfections that aggravate the damage. This rapid transition from infection to structural damage, especially in patients with *STAT3* mutations, highlights the importance of early and aggressive intervention. Managing reinfections after structural lung damage is essential to prevent further deterioration and to improve long-term outcomes.

**Supplementary Information:**

The online version contains supplementary material available at 10.1186/s13023-025-03749-6.

## Introduction

The hyper-IgE syndromes (HIES) comprise a group of rare primary immunodeficiency disorders characterized by a triad of increased infection susceptibility, eczema, and raised serum IgE. Patients with HIES presented with recurrent skin and lung infections after birth. Major causal variants are dominant-negative variants in the signal transducer and activator of the transcription 3 (*STAT3*) gene [1–4].

Close approximate incidence of HIES incidence is unknown, but it is estimated to range from 1 in 500,000 to 1 in 100,000 [5–7]. Due to the rarity of this disease and the lack of widespread genetic testing technology in earlier years, patients often experience delayed diagnosis, sometimes not being diagnosed until adulthood. By this time, patients may already have developed severe pulmonary complications, including secondary severe infections on the basis of structural lung disease, which is their primary cause of mortality [8].

Pulmonary complications are the primary factors affecting the quality of life and long-term prognosis in HIES patients [9]. Our understanding of pulmonary disease in HIES continues to evolve. Previously, it was believed that pulmonary disease in patients with HIES could be attributed to multiple factors, with the most significant cause being parenchymal lung damage due to frequent and severe infections [9]. Other causes include autoinflammation driven by a dysregulated immune system [10]. Recent research suggests that we may have underestimated the significant pulmonary susceptibility and impaired post-injury repair caused by primary pulmonary abnormalities [11, 12]. Overall, research on pulmonary disease in HIES is limited, and the underlying mechanisms of structural lung damage remain unclear.

Early identification is essential for patients with HIES. The first case reports date back to 1966 [13], and HIES awareness has been gradually increasing, facilitating early detection of the syndromes. Most patients are diagnosed during childhood, leading to better prognosis [14]. However, in China, recognition of this disease came relatively late, with the first cases reported in 1984 [15]. Many patients are only definitively diagnosed in adulthood, and prolonged diagnosis is associated with poorer outcomes. Researches have predominantly focused on pediatric patients [16, 17], with only one study addressing adults who received a delayed diagnosis [18]. In many developing countries, HIES are still not widely recognized, and genetic testing technology has yet to be adopted in wider ranges, resulting in a significant number of adults with delayed diagnoses that represent a critical subgroup within the HIES patient population.

We aim to gain insight into the natural course of HIES and the progression of pulmonary disease through a review of the literatures and summarizing adult HIES patients in our study. This will improve our understanding of pulmonary diseases in HIES and aid in better management of these patients.

## Method

From January 2016 to October 2023, ten patients aged 18 years or older with HIES hospitalized at Peking Union Medical College Hospital (PUMCH) were included in our study. The diagnosis of HIES is based on the diagnostic scoring system established by National Institutes of Health (NIH) in 1999, which utilizes 19 clinical and laboratory criteria [19]. A score of greater or equal to 40 confirms the diagnosis of HIES. Also, Whole-exome sequencing was performed on each patient. Patients need to conform with NIH criteria and have a defect in *STAT3* gene in order to be admitted into the database. Pathogenic variants were identified through curated databases (ClinVar [20], OMIM [21], HGMD Pro [22]), with exclusion of variants showing MAF ≥ 0.0001% in control populations (ExAC/gnomAD, 1000 Genomes). Variant pathogenicity was predicted using silico predictive algorithms including SIFT [23], PolyPhen-2 [24], CADD [25], and MutationTaster [26] with default thresholds. The American College of Medical Genetics and Genomics (ACMG) classification followed the 2015 guidelines [27]. The clinical data, including patient demographics, clinical presentations, laboratory results, pathological and radiological examinations, treatment protocols, and follow-ups, were collected. In our study, pulmonary structural damage refers to irreversible damage to the lung parenchyma and interstitium caused by various reasons, including bronchiectasis, pulmonary fibrosis, pneumatocele, multiple cavities, lung destruction, and among other structural changes in the lungs. This study was approved by the Institutional Review Board of Peking Union Medical College Hospital (PUMCH) and conducted in compliance with the Declaration of Helsinki.

We conducted our systematic review using Preferred Reporting Items for Systematic Reviews and Meta-Analyses (PRISMA) [28]. We searched Medline, Embase, and Pubmed databases for all articles about HIES and selected published articles involving humans and written in English between 1966 and 2023. The keywords used were the following ones: “Job Syndrome”[Mesh], “Hyper-IgE Syndromes,” “Hyper-IgE Syndrome,” etc. The specific search strategy is attached. Articles were then assessed for eligibility. Ultimately, we reviewed the pulmonary conditions of patients reported in all previous literature and summarized findings from cohorts focused on pulmonary disease.

## Results

### Demographic features

Our study involved 10 adult patients diagnosed with HIES, all of Chinese descent, including 4 females and 6 males, with a median age of 28.1 years old. HIES diagnosis was confirmed using the NIH scoring system, and *STAT3* mutations were identified in all the patients. These individuals were presenting onset symptoms at birth and diagnosis at a median age of 23 years. The prolonged period from onset to diagnosis, averaging 23 years, underscores a widespread issue of delayed diagnosis.

### Clinical manifestations

The clinical spectrum of HIES encompasses both immunological and non-immunological symptoms (Table 1). In our cohort, immunological manifestations predominantly included eczema (100%, 10/10), skin infections (100%, 10/10), and pneumonia (90.0%, 9/10). Non-immunological symptoms were varied, featuring retained primary teeth (42.9%, 3/7), scoliosis (33.3%, 3/9), fractures with minor trauma (50.0%, 4/8), midline anomaly (11.1%, 1/9), hyperextensibility (20.0%, 1/5), characteristic face (88.9%, 8/9), increased nasal width (62.5%, 5/8), and high palates (50.0%,3/6). Laboratory analyses consistently showed elevated IgE levels (100%, 10/10) and an average eosinophil count of 1.52 × 10^9^cells/L (± 2.65). Notably, two patients developed finger clubbing due to chronic pulmonary issues.

**Table 1:** Clinical features and laboratory parameters of 10 adult patients with STAT3-HIES in PUMCH

Pts	Family history	Retained primary teeth	Pneumonia	Parenchymal lung anomalies	Upper respiratory infections	Scoliosis	Fractures with minor trauma	Midline anomaly	Newborn rash	Eczema	Skin abscesses
1	-	-	+	+	> 6	-	+	-	+	+	+
2	-	-	-	+	0	-	+	-	-	+	+
3	-	+	+	+	> 6	+	-	+	+	+	+
4	+	-	+	+	4–5	+	+	-	+	+	+
5	-	-	+	+	NA	-	-	-	+	+	+
6	+	NA	+	+	3–5	+	NA	-	NA	+	+
7	-	+	+	+	3–4	-	-	-	+	+	+
8	-	NA	+	+	NA	-	+	-	-	+	+
9	-	+	+	+	> 6	-	-	-	+	+	+
10	-	NA	+	-	NA	NA	NA	NA	NA	+	+
Total(%)	2/10 20.0	3/7 42.9	9/10 90	9/10 90	6/7 85.7	3/9 33.3	4/8 50.0	1/9 11.1	6/8 75.0	10/10 100	10/10 100

Pts	Candidiasis	Other serious infections	Hyperextensibility	Lymphoma	Characteristic face	Increased nasal width	High palate	Highest serum-IgE level	Highest eosinophil count	NIH score
1	-	-	-	-	-	-	-	> 5000	1.55	55
2	-	+	-	-	+	-	+	> 5000	0.95	51
3	-	+	+	-	+	+	-	> 5000	0.64	71
4	+	-	-	-	+	+	+	> 5000	1.58	61
5	+	-	-	+	+	-	-	> 5000	1.49	59
6	+	+	NA	-	+	+	NA	> 5000	1.33	68
7	+	-	NA	-	+	NA	NA	> 5000	3.96	57
8	+	+	NA	-	+	+	NA	> 5000	14.24	51
9	+	+	NA	-	+	+	+	> 5000	0.50	64
10	+	+	NA	-	NA	NA	NA	> 5000	3.60	48
Total(%)	7/10 70.0	6/10 60.0	1/5 20.0	1/10 10.0	8/9 88.9	5/8 62.5	3/6 50	10/10 100	1.52 ± 2.65	58.5 ± 7.15

*NA* not available, “ + ”: positive, “-”: negative, pts: patients

Upper respiratory infections: the number of upper respiratory per year

Highest serum-IgE level (IU/ml): Normal < 60 IU/ml

Highest eosinophil count (× 10^9^cells/L): Normal < 0.5 × 10^9^cells/L

Midline anomaly: For example, cleft palate, cleft tongue, hemivertebrae, other vertebral anomaly, etc.

### *STAT3* mutation

Genetic analysis identified six patients with established pathogenic variants (c.1145G > A, c.1907C > T, c.1387_1389delGTG, c.2137G > T, c.1144C > T, c.1909G > A; ClinVar-confirmed, absent in ExAC/gnomAD MAF < 0.0001%), three with hotspot variants (c.1850C > T, c.994C > A, c.2111_2112insC; ExAC/1000 Genomes MAF < 0.001%), and one novel variant (c.53 T > C; gnomAD-absent, 4/4 algorithm concordance). All variants met ACMG criteria: established variants were classified as *pathogenic* (PS1), while hotspot and novel variants were classified as *likely pathogenic* (PM1, PM2, and PP3).

## Pulmonary features of HIES

### Overview of pulmonary involvement

Among all the patients, 9 has lung tissue destruction and the incidence of pneumonia was 90.0% (9/10). Of these 9 patients, only one patient was found to have a pulmonary cyst without pneumonia and to have underwent surgical resection with no pneumonia occurring postoperatively. The other 8 patients all developed varying degrees of pulmonary disease, notably in the form of recurrent pneumonia during the course of the disease and with pulmonary structural damages including bronchiectasis and pneumatocele. These 8 patients typically experienced pulmonary infections within the first 10 years of life (6/8), with 5 cases occurring immediately after birth. In the early stages of the disease, common infections included *Staphylococcus aureus*/ Methicillin-resistant *Staphylococcus aureus* (MRSA) and *Haemophilus influenzae*, while later stages saw infections by *Aspergillus*/fungus, *Pseudomonas aeruginosa*, and *Mycobacterium tuberculosis*. Of these patients, one ( #10) had mild pulmonary disease, caused by *Mycobacterium tuberculosis* infection. After anti-infective treatment, the condition of this patient improved without pulmonary structural damage, and the disease remained stable.

### Microbiology assessment

In the early stages of repeated lung infections, *Staphylococcus aureus* was the most common pathogen, accounting for 90.0% (9/10) of cases. As repeated lung infections led to structural lung damage, the pathogenic spectrum gradually shifted to include *Aspergillus* species and other fungi (70.0%, 7/10), *Mycobacterium tuberculosis* (50.0%, 5/10), and *Pseudomonas aeruginosa* (40.0%, 4/10). Other identified pathogens included *Streptococcus pyogenes* (10.0%, 1/10), *Haemophilus influenzae* (10.0%, 1/10), *Escherichia coli* (10.0%, 1/10), and *Flavobacterium indologenes* (10.0%, 1/10). Other infection-related conditions have also been summarized in Table 2.

**Table 2:** Demographic features and Summary of infection-related conditions in 10 adult patients with STAT3-HIES in PUMCH

		Age									
Pts	Gender	Status	Onset	Diagnosis	First pneumonia	Pulmonary structural damage	APM infections	Onset → Dignosis	First pneumonia → PST*	PST* → APM infections:	*STAT3* Mutation
1	Male	26y	Birth	22y	Birth	7y (P + B)	—	22y	7y	—	c.1145G > A, p.R382Q
2	Female	20y	6 m	15y	—	13y (P)	—	14.5y	—	—	c.1907C > T, p.S636F
3	Male	37y	Birth	28y	Birth	27y (B + P)	33y	28y	27y	6y	c.1850C > T, p.A617V
4	Male	26y	Birth	24y	10y	10y (B + P)	26y	24y	0	16y	c.994C > A, p.H332N
5	Female	28y	Birth	18y	10y	10y (B + P)	15y	18y	0	5y	c.1387_1389delGTG,
											p.V463del
6	Female	32y	Birth	30y	Birth	27y (B)	27y	30y	27y	0	c.2137G > T, p.V713L
7	Male	21y	Birth	15y	Birth	15y (B + P)	15y	15y	15y	0	c.1144C > T, p.R382W
8	Male	52y	32y	47y	32y	32y (B + P)	40y	15y	0	8y	c.2111_2112insC,
											p.Y705Tfs*19
9	Female	36y	Birth	36y	14y	14y (B + P)	35y	36y	0	21y	c.1909G > A, p.V637M
10	Male	23y	18y	21y	18y	—	18y	3y	—	—	c.53 T > C, p.L18P
Total	4/6 (F/M)	28.1 ± 9.4y	0 ± 0.5y	23 ± 12y	10 ± 16y	14 ± 8.5y	26.5 ± 17.5y	22 ± 6.5y	0 ± 3.5y	5 ± 3y	

	Infection					
Pts	Age	type	Microbiology	Therapy	Prophylactic antibiotics	Fellow up
1	childhood	Skin/Lung	*S. aureus*	Antibiotics + Drainage + Surgery	Yes	Stable
2	childhood	Skin/Middle ear/Sinus	*S. pyogenes*	Antibiotics + Drainage	Yes	Stable
	adolescence	Liver/Abdominal cavity	MRSA	Antibiotics + Drainage + Surgery		
3	childhood	Skin/Lung/Middle ear/Sinus/Mastoid	*S. aureus*	Antibiotics + Drainage + Surgery	Yes	Stable
	adolescence	Kidney	*S. aureus*	Antibiotics + Surgery		
	adulthood	Lung	*M. tuberculosis*	Antibiotics		
4	childhood	Skin/Lung	MRSA	Antibiotics + Drainage	Yes	Not contral
	adulthood	Lung/Sinus	*P. aeruginosa*/*Aspergillus*			
5	childhood	Skin/Lung	*S. aureus*/*E. coli* /*P. aeruginosa*	Antibiotics + Surgery	No	died of severe pulmonary infection
	adolescence	Lumbar vertebra/Lung	*Aspergillus*/*M. tuberculosis*	Antibiotics		
6	childhood	Skin/Lung	*P. aeruginosa*	Antibiotics	No	Stable
	adulthood	Mediastinum/Endocardium	Fungus			
7	childhood	Skin(BCG)/Lung	*M. tuberculosis*	Antibiotics	No	Lost to follow-up
	adolescence	Lung	*S. aureus*/*Aspergillus*/*F. indologenes*	Antibiotics + Drainage		
8	adulthood	Skin/Lung/Lumbar vertebra	*S. aureus*/*M. tuberculosis*/ *Aspergillus*/*H. influenzae*	Antibiotics + Surgery	Yes	Lost to follow-up
9	childhood	Skin/Lung	*S. aureus*/*P. aeruginosa*	Antibiotics + Drainage	No	Not contral
	adulthood	Lung/Esophagus	*Aspergillus*/fungus	Antibiotics + Embolotherapy		
10	adulthood	Skin/Lung/Encephalic	*M. tuberculosis*/*Aspergillus*	Antibiotics	No	Stable
Total		Skin (100%, 10/10) Lung (90.0%, 9/10) ENT (30.0%, 3/10) Lumbar vertebra (20.0%, 2/10) Encephalic, Endocardium, Mediastinum, Liver, Kidney, Abdominal cavity, Esophagus (10.0%each, 1/10)	*S. aureus* /MRSA (80.0%, 8/10) *Aspergillus*/fungus (70.0%, 7/10) *M. tuberculosis* (50.0%, 5/10) *P. aeruginosa* (40.0%, 4/10) *S. pyogenes*, *H. influenzae*, *E. coli*, *F. indologenes* (10.0%each, 1/10)	Antibiotics (100%, 10/10) Drainage (60.0%, 6/10) Surgery (50.0%, 5/10) Embolotherapy (10.0%, 1/10)	Yes (50.0%,5/10) No (50.0%,5/10)	Died (10.0%,1/10) Stable (50.0%, 5/10) Lost to follow-up (20.0%, 2/10) Not contral (20.0%, 2/10)

Age ranges are defined as 0–10 years (childhood), 6–22 years (adolescence), and 20–34 years (adulthood). Total values are summarized for each age range

*NA* not available, pts:patients, *F* female, *M* male, *y* year, *m* month, *y* years old, *MRSA* Methicillin-resistant *Staphylococcus aureus, BCG* Bacillus Calmette-Guérin, *ENT* Ears, Nose and Throat, *B* bronchiectasis; *P* pneumatoceles

*Escherichia coli: E. coli*, *Flavobacterium indologenes: F. indologenes*, *Haemophilus influenzae: H. influenzae*, *Mycobacterium tuberculosis: M. tuberculosis*, *Pseudomonas aeruginosa: P. aeruginosa*, *Streptococcus pyogenes: S. pyogenes*, *Staphylococcus aureus*: *S. aureus*

PST*: Pulmonary structural damage; APM infections: *Aspergillus*/fungus/*P. aeruginosa*/*M. tuberculosis* infections

### Radiological imaging assessment

**Fig. 1:**
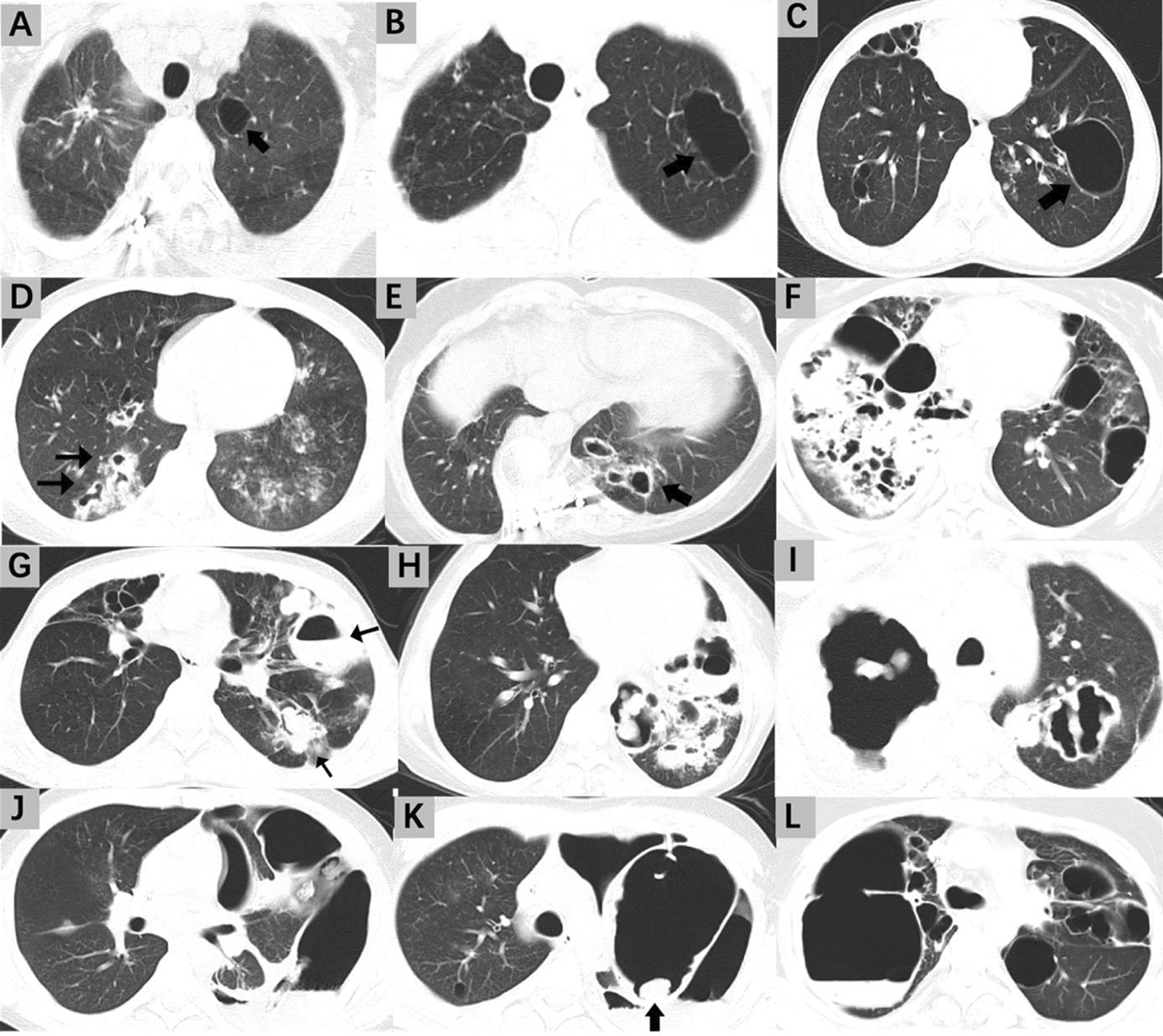
Chest CT of characteristic lung damage of parenchyma and airways in adult patients with STAT3-HIES. **A** (Patient #6): Thin-walled cysts in the left upper lobe. **B**&**C** (Patient #4): Large pneumatoceles in the left upper lobe. **D** (Patient #7): Ground-glass opacities and marked bronchiectasis with mucoid impactions. **E** (Patient #6): Central bronchiectasis and visible dilatation of the airways. **F** (Patient #5): Multiple cavitary lesions with thickened walls, marked bronchiectasis, and surrounding consolidation, indicative of CCPA. **G** (Patient #4): Bronchiectasis with a finger-in-glove sign, indicating bronchial impaction with mucus and air-fluid levels, suggestive of ABPA. **H** (Patient #9): Cavitary lesions with thick posterior walls and air-fluid levels. **I** (Patient #8): Extensive cavitation with thick posterior walls. **J** (Patient #7): Pneumothorax with mediastinal shift, secondary to a large ruptured pneumatocele or bulla. **K** (Patient #7): Giant bulla with significant compression of adjacent lung tissue; there is a centrally located aspergilloma within one of the cavities. **L** (Patient #5): Multiple pneumatoceles and thick-walled cystic spaces, indicating severe structural lung damage

All 8 patients (#1, #3, #4, #5, #6, #7, #8, #9) with recurrent pneumonia showed obvious pulmonary structural damage (80.0%, 8/10). Chest X-ray (CXR) and computerized tomography (CT) assessments revealed lung parenchyma and airway destruction. Recurrent pulmonary infections led to airway remodeling with bronchial wall-thickening, bronchiectasis, mucus-plugging, mosaic perfusion, and expiratory air-trapping. Cystic parenchymal abnormalities included cysts, pneumatoceles, and cavities; these abnormalities arose during infection and often persisted. Frequent parenchymal and airway remodelling are presented in Fig. 1.

The spectrum of CT findings in pulmonary aspergillosis associated with HIES includes (1) aspergillomas (#6, #7, #8), where fungal balls are found within pre-existing lung cavities such as bronchiectasis or pneumatoceles, (2) chronic cavitary pulmonary aspergillosis (CCPA) (#4, #5, #7, #8, #9) characterized by cavity wall thickening, fungal balls, and peri-cavitary infiltrates, and (3) an allergic bronchopulmonary aspergillosis (ABPA) (#4, #8, #9) -like presentation with bronchiectasis and mucoid impactions that resemble toothpaste-shaped or finger-in-glove opacities. Additionally, mixed patterns and occurrences of invasive aspergillosis can also be observed.

Additionally, one patient (#2), without any signs of infection, was incidentally found to have a pneumatocele, which was surgically removed. Seven years after the surgery, there have been no occurrences of pneumonia or further pulmonary structural damage.

### Histological features

**Fig. 2:**
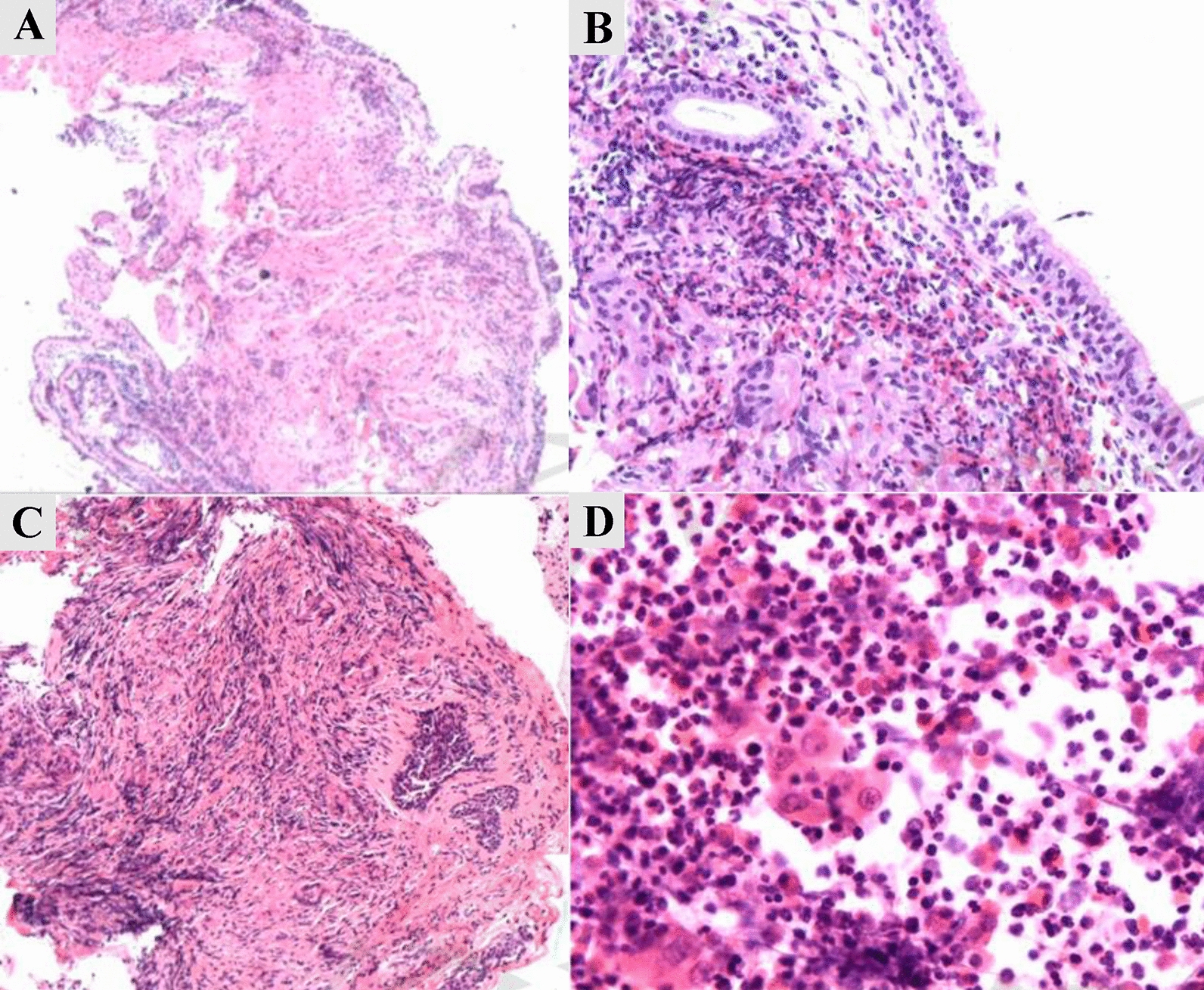
Histological Features of Pulmonary disease in STAT3-HIES. **A** (HE, 10x): Inflammatory exudate and mild chronic inflammation of the bronchial mucosa (#8, bronchial mucosa), with scattered eosinophil infiltration. **B** (HE, 20x): Substantial infiltration of inflammatory cells in the submucosa of the bronchus (#8, anterior segment of the left upper lobe), with granuloma formation, multinucleated giant cells, and necrosis. Special staining suggests a fungal infection. **C** (HE, 10x): Extensive infiltration of inflammatory cells in the submucosa of the bronchus (#8, left main bronchus), with granuloma formation, multinucleated giant cells, and necrosis. **D** (HE, 40x): Neutrophils, eosinophils, and ciliated columnar epithelial cells in bronchial brushings (#4, bronchoscopy brushings)

In our patients, four cases (#1, #4, #7, #8) underwent bronchoscopy to obtain histological results, one case (#2) had postoperative histology results from surgical resection of a lung bulla, and two cases (#6, #10) had histology obtained via percutaneous needle biopsy (Fig. 2). No tumor cells were observed in any of the samples (100%, 7/7). Detailed descriptions were available for five cases. Some presented with acute inflammation (40%, 2/5), while others showed chronic inflammation/granuloma formation (80%, 4/5). Eosinophils were observed in all cases (100%, 5/5). Fungal hyphae were noted in one case (#6), and special staining was positive in one case (#8).

### Course of pulmonary involvement

We summarized the timeline of disease development in 9 patients with pulmonary structural damage detected by imaging (Table 2). Most patients (77.8%, 7/9) had onset after birth, with the age of initial pneumonia occurring at 5 years (± 5y), age of *Staphylococcus aureus* infection at 10 years (± 7y), median age of pulmonary structural damage at 14 years (± 8.5y), age of combined *Aspergillus*/fungus*, Pseudomonas aeruginosa*, and *Mycobacterium* infections at 27 years (± 6.75y), age of definite diagnosis at 24 years (± 6y), and age of surgery at 13 years (± 7.25y). The average time from onset to definitive diagnosis of these 9 patients was 22 years, and time from initial pneumonia to the identification of pulmonary structural damage was 0 year, with time from structural damage to *Aspergillus*/fungus infection averaging 5 years. Thus, these patients had onset before the age of 10 years with early-stage infections mainly caused by *Staphylococcus aureus* and pulmonary structural damage detected almost simultaneously with the occurrence of infection. After approximately 5 years, infections by *Aspergillus*/fungus*, Pseudomonas aeruginosa,* and *Mycobacterium tuberculosis* gradually appeared.

### Treatment of pulmonary involvement

Pulmonary treatment primarily targets lung infections and structural lung diseases. During acute lung infections, broad-spectrum antibiotics are the mainstay of treatment. In patients with structural lung disease (such as bronchiectasis and pulmonary cysts), antibiotic coverage for *Pseudomonas aeruginosa* and *Aspergillus* species is necessary. For prophylactic antibiotic therapy, sulfamethoxazole is chosen for the most common pathogen, *Staphylococcus aureus.* In the presence of structural lung disease, itraconazole is used to prevent fungal infections.

Additionally, in cases of CCPA and ABPA, the duration of antibiotic therapy needs to be extended. Among our patients, 100% (10/10) received antibiotics, with 50.0% (5/10) receiving prophylactic antibiotics. Antifungal treatment – itraconazole, voriconazole, or amphotericin B – was administered along with extended antibiotic therapy to 7 patients with *Aspergillus*/fungus infections. Other treatments included local drainage, surgery, interventional procedures, intravenous immunoglobulin (IVIG), vaccination, and regular pathogen monitoring. In our cohort, 60.0% (6/10) of the patients underwent drainage, 50.0% (5/10) of the patients had surgical interventions, and 10.0% (1/10) of the patients received interventional embolization treatment. IVIG use in HIES remains controversial. While some evidence suggest benefits for patients with low immunoglobulin levels or poor vaccine responses [29, 30], overall, it shows only mild efficacy, mainly in HIES patients with eczema [31], and its high cost, potential side effects, and limited accessibility prevent it from being a standard treatment for HIES, though it may help those with severe lung disease [7]. Thus, we did not routinely use IVIG. However, during follow-up, we observed that some patients attempted treatments with limited evidence, such as IVIG. We provided chest physiotherapy (in the form of postural drainage and percussion) and airway clearance therapies to 8 patients with structural lung disease. None of our patients underwent lung transplantation.

## Treatment of extrapulmonary involvement

The treatment of our HIES patients adhered to the following principles, focusing on both pulmonary and extrapulmonary aspects. Extrapulmonary treatment included anti-infective therapy for other deep-seated infections. Among our patients, 70.0% (7/10) had infections outside of the skin and respiratory tract, including the lumbar spine, intracranial, endocardium, mediastinum, liver and abdomen, kidneys, and esophagus. All of these patients were treated with antibiotics, and surgery and drainage were performed in 4 cases. Other treatments included skin (for eczema, staphylococcal infections, and mucocutaneous candidiasis), skeletal and connective tissue (vitamin D supplementation, scoliosis monitoring), oral and dental care (regular check-ups, extraction of delayed deciduous teeth), vascular monitoring (regular aneurysm screening), treatment of secondary lymphomas, and genetic counselling, which will not be discussed in detail here. Stem cell transplantation remains controversial, as transplantation's long-term efficacy and cost-effectiveness for HIES patients remain inadequately established in current research. We did not performed any such transplants.

## Follow-up and prognosis

Of the 10 patients monitored, 5 have achieved a stable condition, 2 continue to necessitate recurrent interventions for persistent infections, 1 developed lymphoma and ultimately succumbed to severe pulmonary infection with septic shock during treatment, and 2 were lost to follow-up.

## Literature review and summary

### Case and cohorts of HIES

A total of 1,367 articles were retrieved (Figure.S1). Among the articles were 232 cohort studies and 573 case reports. Out of all cohorts, there were 16 national-level large cohorts [7, 8, 14, 16, 17, 32–42], with one cohort lacking descriptions of pulmonary disease [32]. Pulmonary complications varied across different countries and regions. The summaries of the pulmonary complications in the other 15 cohorts focused primarily on the incidence of pneumonia, pulmonary cysts, and bronchiectasis (Table S1). The prevalence of pulmonary structural damage is generally higher, with a range of 15.0% to 65.0% for bronchiectasis and a range of 16.7% to 68.4% for pneumatocele. Among them, the prevalence of bronchiectasis in HIES patients with *STAT3* mutation is 65.0%, and pneumatocele is 52.0%.

### Studies of pulmonary disease in HIES

Eight out of all cohort studies focused specifically on pulmonary disease, and the summary of them included pulmonary complications, pathogens, surgical interventions, and treatments (Table S2) [8, 43–49].

### Pulmonary disease of different gene variants

Recently, several new causative variants have been identified, including *PGM3*, *ZNF341*, *CARD11*, *IL6ST*, *IL6R*, *TGFBR1/2*, *ERBB2IP*, and *SPINK5* [40, 50–55]. We compared the incidence of pulmonary complications among HIES patients with other genetic mutations (Table S1). For IL6ST mutation, prevalence of bronchiectasis and pneumatocele are 60.0% and 54.5%. For *ZNF341* mutation, they are 56.3% and 35.3%. However, *TGFBR* mutation has been reported in only 10.0% of patients with pneumatocele, and no pulmonary structural damage has been described. Among HIES patients, those with *STAT3* mutations are more likely to experience pulmonary structural damage, particularly pneumatocele formation.

### Three stages of pulmonary disease in HIES

**Fig. 3:**
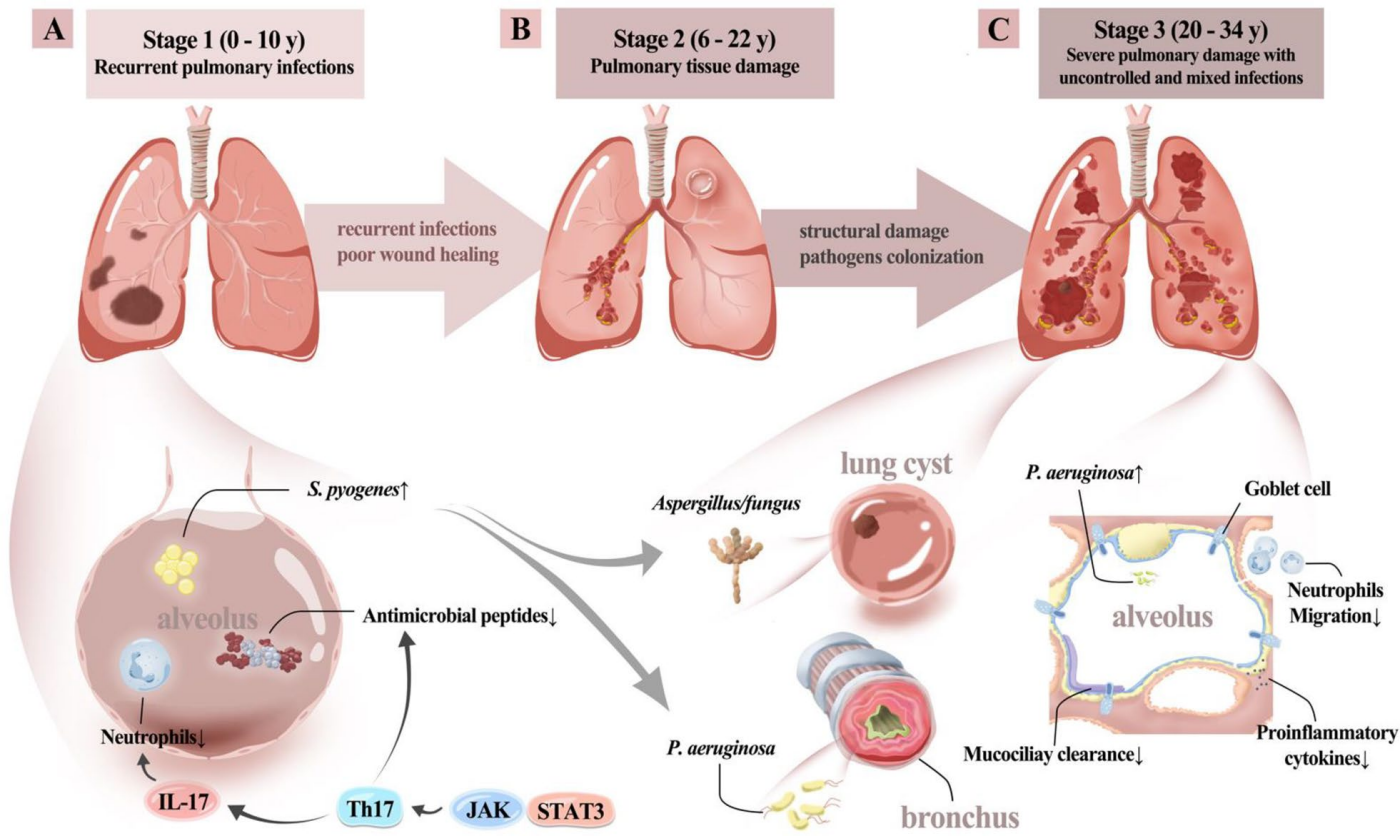
Three stages of pulmonary disease in adult patients with STAT3-HIES and the potential pathogenesis. **A**. Stage 1: Recurrent pulmonary infections (0–10 years old), patients with STAT3-HIES exhibit a defect in the JAK-STAT signaling pathway, leading to impaired differentiation and function of Th17. This affects neutrophil chemotaxis and proliferation. Also, it reduces the production of antimicrobial peptides, as respiratory epithelial cells and keratinocytes rely on IL-17 to produce these peptides. Ultimately, this defect increases susceptibility to *Staphylococcus aureus* infections, primarily affecting the skin and respiratory tract. Patients in early childhood often present with recurrent respiratory infections. **B**. Stage 2: Pulmonary tissue damage (6–22 years old), repeated infections cause pulmonary tissue damage due to significant pulmonary susceptibility and impaired post-injury repair resulting from primary pulmonary abnormalities. This cycle of recurrent infections progressively damages lung tissue, while impaired repair mechanisms lead to permanent structural changes. As this cycle continues, pulmonary damage worsens, making patients more prone to develop bronchiectasis and pulmonary cysts. **C**. Stage 3: Severe pulmonary damage with uncontrolled and mixed infections (22–34 years old), patients enter a malignant cycle of pulmonary structural damage and infection, with a shift in pathogens towards predominantly mixed infections involving *Aspergillus*/fungus, *Pseudomonas aeruginosa*, and *Mycobacterium tuberculosis*. Additionally, patients exhibit abnormal differentiation of airway epithelium, characterized by decreased ciliated cells and increased goblet cells and club cells, resulting in excessive mucus secretion. These changes facilitate pathogen adherence to the local airway surfaces. Furthermore, during this process, there is a reduction in proinflammatory cytokines, collectively exacerbating the risk of infection. Severe structural abnormalities of the lungs and uncontrollable infections often develop in this stage

Through a review of all previously published cases, the course of pulmonary disease in HIES patients can be generally divided into three stages (Fig. 3). The first stage is characterized by recurrent pneumonia (0–10 years old), primarily caused by *Staphylococcus aureus*. The second stage involves pulmonary structural damage (6–22 years old), including abscesses, cavity formation, bronchiectasis, and pneumatoceles. The third stage enters a malignant cycle of pulmonary structural damage and infection (20–34 years old), with a predominant shift in pathogens to mixed infections of *Aspergillus*/fungus, *Pseudomonas aeruginosa*, and *Mycobacterium tuberculosis*. It is noteworthy that the time from the initial pneumonia to pulmonary structural damage is very short, with a median time of 0 years (± 3.5y). Additionally, the average time from pulmonary structural damage to the onset of *Aspergillus*/fungus infection is 5 years.

## Discussion

HIES are a heterogeneous group of inborn errors of immunity-sharing manifestations that include increased infection susceptibility, eczema, and raised serum IgE [56]. Our research presents significant deviations from previous reports, particularly with regard to the onset and diagnosis ages of patients. Former large case series referenced in the literature report onset ages at 0 ± 1.5 years and diagnosis ages at 24 ± 8 years. Our cohort, drawn exclusively from the adult department, includes several individuals whose disease onset occurred in adulthood, with the rest experiencing a severe diagnostic delay. The average duration from disease onset to diagnosis spanned 20 years, with the longest case extending up to 36 years. Many patients exhibited symptoms shortly after birth and underwent numerous medical consultations due to recurrent infections, leading to a convoluted path to diagnosis. The delay in diagnosis, aside from HIES's low incidence rate and the lack of adequate recognition among clinicians, is also attributed to the cumulative nature of clinical presentations as they intensify with age, such as repeated infections, retention of primary teeth, and scoliosis. It is often only after the emergence of unexplainable severe infections that HIES is identified. Moreover, even within the same mutation, HIES patients exhibit significant clinical phenotype heterogeneity, which calls for further investigation into the underlying mechanisms.

The selection bias in our study's inclusion criteria provided us with opportunities to observe findings not commonly reported in previous case series. Our cases often involved long disease courses, with patients presenting with recurrent severe infections, particularly in the lungs, and a considerable number developing severe pulmonary complications, indicating a relatively advanced stage of the disease. By reviewing the disease progression in these patients, we have gained insights into a more complete progression pattern of pulmonary disease in HIES. The pathogenic genes of HIES are not limited to *STAT3*; however, its main pathogenic mechanisms and gene mutations still revolve around the JAK-STAT pathway. Since all patients in our study exhibited *STAT3* gene mutations, our discussion will primarily focus on STAT3-HIES.

By summarizing our case series and reviewing previous case series, we have characterized the pulmonary disease trajectory of these patients in three stages. The first stage typically presents symptoms from birth to early childhood, with recurrent pulmonary infections predominantly caused by *Staphylococcus aureus*. The second stage usually occurs from childhood to adolescence and is characterized by pulmonary structural damage, including abscesses, cavitation, bronchiectasis, lung cysts, and the onset of mixed infections. The pathogens become increasingly diverse, including *Pseudomonas aeruginosa, Aspergillus*/fungus and *Mycobacterium tuberculosis*. The third stage typically occurs from adolescence to adulthood, entering a malignant cycle of pulmonary infection and structural damage. Pulmonary infections become difficult to control, leading to a progressive decline in lung function. Severe cases may present with massive hemoptysis, pulmonary arterial hypertension, recurrent hospitalizations, significantly reduced quality of life, and some cases may result in death.

From a pathophysiological perspective, the mechanisms of recurrent infections and the aetiology in the first stage are relatively clear. STAT3 is a critical cytoplasmic protein. Its dysregulation results in defects in the Janus kinase-signal transducer and activator of transcription (JAK-STAT) signaling pathway, impairing T helper cell type 17 (Th17) differentiation and function [57–59]. This, in turn, reduces neutrophil proliferation, diminishes chemotactic activity, suppresses inflammation, and heightens vulnerability to bacterial and fungal infections [60–62]. Both keratinocytes and bronchial epithelial cells require combined stimulation from Th17 and classical proinflammatory cytokines to produce anti-Staphylococcal agents, elucidating the prevalence of Staphylococcal infections in the skin and respiratory tract [63]. The production of inflammatory cytokines is compromised, leading to a subtle inflammatory response, which manifests as cold abscesses in some HIES patients [13, 62].

The turning point in the patient's disease course is the destruction of the pulmonary structure. In our study, the average time from pulmonary structural damage to the onset of *Aspergillus*/fungus infection is 5 ± 3 years. In other words, once transitioned from the second stage to the third stage, the microbiology of pulmonary infections in patients quickly changes from a relatively simple *Staphylococcus aureus* infection to mixed infections, including *Pseudomonas aeruginosa, Aspergillus*/fungus and *Mycobacterium tuberculosis*. This leads to the onset of a malignant cycle of pulmonary infection and structural damage, resulting in progressive deterioration of the condition.

For HIES patients, the known clear cause of pulmonary structural damage is recurrent infections. However, our observational study results have revealed phenomena that cannot be explained solely by this cause: (1) The time from initial pneumonia to the appearance of pulmonary structural damage is very short, with a median time of 0 (-16 ~ 10)year. (2) Our observations revealed that HIES patients had a higher proportion of cystic lesions compared to other immunodeficiency disorders with recurrent pulmonary infections. (3) We noted that pulmonary complications, such as pneumatocele, could occur not only during acute infections but also in non-infectious periods. (4) In patients with other genetic mutations, the occurrence of pulmonary structural damage, especially pneumatoceles, is less frequent, while in *STAT3* mutation patients, it accounts for 80.0%. Collectively, these findings lead us to postulate that there are unique pathophysiological mechanisms behind pulmonary structural damage, particularly cystic lesions. Based on these findings, we hypothesize that there may be unique pathophysiological mechanisms underlying pulmonary structural damage, particularly cystic lesions, in patients with STAT3-HIES. Previous researches have suggested several potential mechanisms that may contribute to this phenomenon.

The first potential mechanism underlying pulmonary structural damage in HIES patients is impaired lung tissue repair. Studies using mouse models with selective respiratory epithelial STAT3 deficiency have demonstrated that STAT3-dependent repair of bronchiolar and alveolar epithelium is critical for maintaining lung tissue integrity [64]. Hokuto et al. discovered that mice with STAT3 deficiency in respiratory epithelial cells experienced accelerated lung injury, abnormal alveolocapillary integrity, surfactant deficiency, and poor lung mechanics, leading to epithelial and vascular damage [65]. The second potential mechanism involves the impact of *STAT3* mutations on airway epithelial function. Research by Zhang et al. suggested that *STAT3* mutations reduce STAT3 protein phosphorylation, nuclear translocation, transcription activity, and protein stability in airway basal cells [12].

As a consequence, STAT3-mutated airway basal cells give rise to airway epithelial cells with abnormal cellular composition and loss of coordinated mucociliary clearance. Notably, HIES STAT3 airway epithelial cells are defective in bacterial killing and fail to initiate vigorous proinflammatory responses and neutrophil transepithelial migration [12]. These findings all suggest that in addition to impaired lung tissue repair following infection-induced injury, HIES patients possess inherent mechanical and inflammatory defects in the respiratory epithelium.

Moreover, an unexpected finding in our study was the incidental discovery of a pulmonary bulla in a patient with a *STAT3* mutation despite the absence of pulmonary infection. In patients with HIES, due to abnormalities in the JAK-STAT pathway, the inflammatory response is suppressed, and clinical manifestations following infections are often mild, making the condition prone to being overlooked or masked. It is highly likely that this particular patient experienced a subclinical or occult pulmonary infection process that went unnoticed. During follow-up, this patient did not exhibit recurrent or severe pulmonary infections or structural lung damage, and the overall pulmonary manifestations were relatively mild. This also suggests that, prior to the onset of severe and obvious infections or structural lung damage, patients may already have occult infections and subtle structural changes in the lungs that go undetected. Therefore, monitoring the condition of HIES patients cannot rely solely on symptoms; objective assessments such as imaging are necessary.

Based on our observational findings and the mechanistic research, the treatment of pulmonary disease in HIES patients may benefit from an expanded focus on targeting epithelial and immune cells. For example, therapies targeting the cystic fibrosis transmembrane conductance regulator (CFTR) modulation for cystic fibrosis, a genetic mutation disease, may offer valuable insights. CFTR modulator therapies aim to enhance the production, intracellular processing, and function of defective CFTR protein [66, 67]. Gene therapy and stem cell-based therapy represent promising avenues for the treatment of genetic diseases. Similarly, inhalable pharmacological activation of the STAT3 signaling pathway may hold the potential for restoring normal airway epithelial cell structure, host defense function, and injury repair.

Further exploration of these therapeutic strategies is warranted. Research into the mechanisms of pulmonary disease in HIES is sparse, particularly investigations beyond infectious etiologies. Given the rarity of HIES, previous studies have been observational, and early-onset pulmonary structural damage has not been addressed in case reports. We have found few related basic research studies.

Therefore, in addition to correcting poorly functioning immune cells, we provide evidence that therapies targeting STAT3-dependent airway epithelial cell abnormalities may be useful in eradicating pathogens and reducing pulmonary complications.

Pulmonary complications are responsible for high morbidity and mortality rates in patients with HIES. Meanwhile, pulmonary symptoms are a heavy burden for these patients. Hence, comprehending the development of pulmonary abnormalities and the mechanisms driving them is essential for the prophylaxis and control of pulmonary disease and critically important for enhancing the survival quality and prognostic outcomes of patients.

By conducting a retrospective analysis of the clinical data from the 10 patients with HIES, with special emphasis on the 9 cases that developed pulmonary complications, we have attained a deeper insight into the clinical phenotype of HIES, specifically concerning the pulmonary manifestations of the disorder.

Our study has several limitations. First, the retrospective nature of the study and the small sample size may introduce selection bias and limit the generalizability of our findings. Second, the lack of comprehensive pulmonary function data limits our ability to fully characterize the functional impact of lung disease in HIES patients. Future studies should address these limitations by incorporating prospective designs, larger cohorts, and systematic functional assessments.

## Conclusions

The progression of pulmonary disease in HIES patients underscores a critical three-step process: initial recurrent infections, development of structural lung damage, and subsequent reinfections that aggravate the damage. This rapid transition from infection to structural damage, especially in patients with *STAT3* mutations, highlights the importance of early and aggressive intervention. Managing reinfections after structural lung damage is essential to prevent further deterioration and to improve long-term outcomes.

## Supplementary Information

Additional file 1.

## Data Availability

All data generated or analysed during this study are included in this published article and its supplementary information files.

## Abbreviations

ABPA: Allergic bronchopulmonary aspergillosis
ACMG: The American college of medical genetics and genomics
CCPA: Chronic cavitary pulmonary aspergillosis
CFTR: Cystic fibrosis transmembrane conductance regulator
CT: Computerized tomography
CXR: Chest X-ray
HIES: Hyper-IgE syndromes
IVIG: Intravenous immunoglobulin
JAK-STAT: The Janus kinase-signal transducer and activator of transcription
MRSA: Methicillin-resistant *Staphylococcus aureus*
NIH: The National Institutes of Health
PRISMA: Preferred reporting items for systematic reviews and meta-analyses
PUMCH: Peking Union Medical College Hospital
*STAT3*: The signal transducer and activator of the transcription 3
Th17: T helper cell type 17
