# Supplementary material for: Pulmonary features and stage of disease in adult patients with hyper-IgE syndrome: a single-centre clinical study and literature review
Source: Orphanet J Rare Dis. 2025 Jun 3;20:270. doi: 10.1186/s13023-025-03749-6 (PMC12131643; doi:10.1186/s13023-025-03749-6)

**Supplementary file**

**Title:** Pulmonary features and stage of disease in adult patients with hyper-IgE syndrome: A single-centre clinical study and literature review.

**Authors:** Tiange Xie^a^, Na Xu^a^, He Zhao^a^, Yingdong Han^a^, Juan Wu^a^, Hong Di, Min Peng^b^, Ting Zhang^b^, Hongwei Fan^c^, Yun Zhang^a*^, Xuejun Zeng^a*^.

a. Department of family medicine & Division of General Internal Medicine, Department of internal medicine. Peking Union Medical College Hospital, Chinese Academy of Medical Sciences, State Key Laboratory of Complex Severe and Rare Diseases (Peking Union Medical College Hospital), Beijing, China.

b. Department of Pulmonary and Critical Care Medicine, Peking Union Medical College Hospital, Chinese Academy of Medical Sciences, Peking Union Medical College, Beijing, China.

c. Department of Infectious Diseases, Peking Union Medical College Hospital, Peking Union Medical College, Chinese Academy of Medical Sciences, Beijing, China.

**Co-Corresponding authors:**

Yun Zhang M.D. Associate professor of medicine.

Xuejun Zeng M.D. PhD professor of medicine.

**E-mail:** zhangyun10806@pumch.cn. zxjpumch@126.com.

Telephone number: +86 010 69156098.

**Address:** Department of Family Medicine & Division of General Internal Medicine, Department of Medicine. Peking Union Medical College Hospital, Chinese Academy of Medical Sciences, State Key Laboratory of Complex Severe and Rare Diseases (Peking Union Medical College Hospital). No. 1 Shuaifuyuan, Dongcheng District, Beijing, 100730, China.

**Table of Contents**

Table S13

Table S24

Fugure S15

|  | HIES |  |  | STAT3-HIES | | | | | | | | | | | | | | | |
| --- | --- | --- | --- | --- | --- | --- | --- | --- | --- | --- | --- | --- | --- | --- | --- | --- | --- | --- | --- |
|  | IL16ST | ZNF341 | Our study | (France, 2012) | (China, 2017) | (USA, 2018) | (Germany, 2018) | (Iran, 2019) | ( Iran, 2019) | (Germany, 2019) | (Italy, 2019) | (France, 2019) | (China, 2020) | (China, 2020) | (India 2021) | (Germany, 2021) | (Israel 2022) | (Tunisia, 2022) |  |
| size | 12 | 20 | 10 | 60 | 17 | 85 | 8 | 4 | 19 | 14 | 28 | 7 | 11 | 20 | 27 | 21 | 3 | 6 |  |
| L% | 91.7 | 56.3 | 90.0 | 90.0 | 100 | 72.0 | 62.5 | 100 | 84.2 | 100 | 60.0 | 42.9 | — | 95. | 63.0 | — | 100 | 83.8 |  |
| B% | 60.0 | 35.3 | 80.0 | 65.0 | 17.0 | 24.7 | 37.5 | 25.0 | 21.1 | 50.0 | 53.4 | — | — | 15.0 | — | — | 100 | — |  |
| P% | 54.5 | 10.0 | 80.0 | 52.0 | 41.2 | 18.8 | 37.5 | 25.0 | 68.4 | 50.0 | 39.4 | 28.6 | — | 50.0 | 26.0 | — | 33.3 | 16.7 |  |

Table S1. Summary of pulmonary complications among adult patients with STAT3-HIES and other HIES

L: Lung infection, P: Pneumonia, B: Bronchiectasis

Table S2. Summary of studies focused on pulmonary disease of HIES

|  | Country | Time of publication | Cohort size | STAT3  mutation | Age of diagnosis | Pneumonia | Bronchiectasis | Pneumatocele | Bronchopleurall fistulae | Aspergillus | P.aeruginosa | S. aureus | TB/NTM | Decreased Lung function | Surgical intervention | Mortality |
| --- | --- | --- | --- | --- | --- | --- | --- | --- | --- | --- | --- | --- | --- | --- | --- | --- |
| 1 | Poland | 2022 | 22 | 7 | 4.4 | 7 | -- | 4 | - | 1 | - | - | - | - | 3 | - |
| 2 | USA | 2018 | 144 | - | - | 127 | 96 | 39 | - | 50 | 43 | 39 | 17 | 75 | - | - |
| 3 | Iran | 2023 | 4 | 4 | 12 | 4 | 4 | 2 | 1 | 3 | - | - | - | - | 4 | 1 |
| 4 | Italy | 2023 | 30 | 30 | 16.6 | 23 | 14 | 13 | - | 4 | - | - | - | - | - | 3 |
| 5 | German | 2018 | 14 | 14 | 22．4 | 13 | 7 | 7 | 4 | 8 | 8 | - | - | 11 | 9 | - |
| 6 | USA | 2013 | 32 | 30 | - | 30 | - | - | - | 10 | - | 7 | - | - | - | - |
| 7 | France | 2019 | 13 | 13 | - | 13 | 8 | 1 | - | 13 | - | - | - | - | 6 | - |
| 8 | ZA | 2018 | 110 | - | 23 | - | 46 | 45 | - | - | - | - | - | - | - | - |

Age of diagnosis: the patient’s age at the time of definitive diagnosis

ZA: North American

Figure S1. Flow chart of the literature search


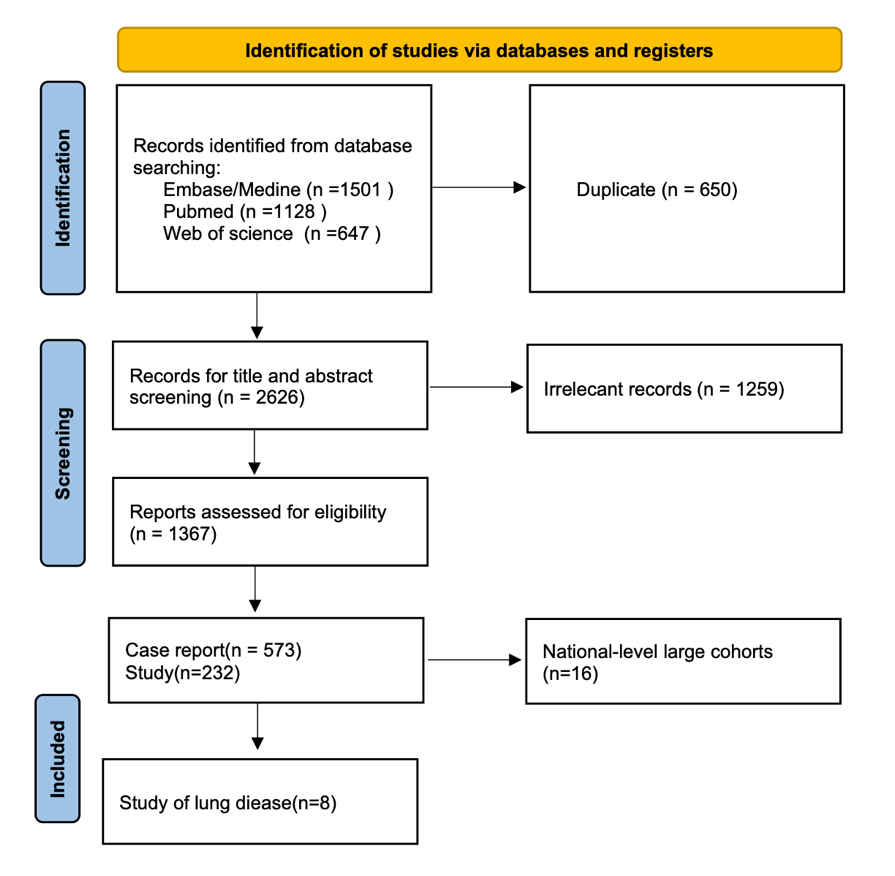

Supplement: Supplementary file 1 — Additional file 1. [file 13023_2025_3749_MOESM1_ESM.docx]
